# Artificial polyhydroxyalkanoate poly[2-hydroxybutyrate-*block*-3-hydroxybutyrate] elastomer-like material

**DOI:** 10.1038/s41598-021-01828-9

**Published:** 2021-11-17

**Authors:** Yuki Kageyama, Hiroya Tomita, Takuya Isono, Toshifumi Satoh, Ken’ichiro Matsumoto

**Affiliations:** 1 Graduate School of Chemical Sciences and Engineering, Hokkaido University, N13W8, Kitaku, Sapporo, 060-8628 Japan; 2 Division of Applied Chemistry, Faculty of Engineering, Hokkaido University, N13W8, Kitaku, Sapporo, 060-8628 Japan

**Keywords:** Biomaterials, Biotechnology, Biosynthesis, Polymer chemistry

## Abstract

The first polyhydroxyalkanoate (PHA) block copolymer poly(2-hydroxybutyrate-*b*-3-hydroxybutyrate) [P(2HB-*b*-3HB)] was previously synthesized using engineered *Escherichia coli* expressing a chimeric PHA synthase PhaC_AR_ with monomer sequence-regulating capacity. In the present study, the physical properties of the block copolymer and its relevant random copolymer P(2HB-*ran*-3HB) were evaluated. Stress–strain tests on the P(88 mol% 2HB-*b*-3HB) film showed an increasing stress value during elongation up to 393%. In addition, the block copolymer film exhibited slow contraction behavior after elongation, indicating that P(2HB-*b*-3HB) is an elastomer-like material. In contrast, the P(92 mol% 2HB-*ran*-3HB) film, which was stretched up to 692% with nearly constant stress, was stretchable but not elastic. The differential scanning calorimetry and wide-angle X-ray diffraction analyses indicated that the P(2HB-*b*-3HB) contained the amorphous P(2HB) phase and the crystalline P(3HB) phase, whereas P(2HB-*ran*-3HB) was wholly amorphous. Therefore, the elasticity of P(2HB-*b*-3HB) can be attributed to the presence of the crystalline P(3HB) phase and a noncovalent crosslinked structure by the crystals. These results show the potential of block PHAs as elastic materials.

## Introduction

Bacterial polyhydroxyalkanoates (PHAs) are biobased polyesters that can be used as commodity plastics^1^ and have attracted considerable interest because of their superior biodegradability in a range of different natural environments, including the sea^2^. In addition, PHAs are used in a variety of potential applications, such as biocompatible materials^3^, components in composites^4,5^, and components in functionalized protein complexes^6^. A key technology for the practical use of PHAs is the regulation of their physical properties. The monomer constituents and their composition play major roles in improving the resistance of the material to impact^7–9^. The regulation of crystallization behavior is also important for the mechanical properties and storage stability^10^.

A powerful strategy to improve the properties of such polymers is to create artificial PHAs that contain unusual monomer constituents^11,12^. 2-Hydroxyalkanoate (2HA)-containing PHAs are particularly useful materials that show properties distinct from those of natural PHAs^13^. It is noteworthy that 2HA-containing PHAs are produced using engineered PHA synthases. Thus, PHA synthases play a central role in the biosynthesis of 2HA-containing PHAs. PhaC1_Ps_STQK was the first discovered PHA synthase that can incorporate a variety of 2HA units, such as lactate, glycolate^14^, 2-hydroxybutyrate (2HB), and amino acid-derived 2HAs^13,15,16^. To date, PhaC1_Ps_STQK, which is a class II enzyme with pairwise point mutations, and homologous enzymes with the same mutations have been used for the biosynthesis of 2HA-containing PHAs^17,18^. We previously reported the class I PHA synthase PhaC_AR_ that can efficiently incorporate 2HB units^19^. PhaC_AR_ is an engineered chimeric enzyme, composed of N- and C-terminal regions of *Aeromonas caviae* and *Ralstonia eutropha* (*Cupriavidus necator*) PHA synthases, respectively^20^. PhaC_AR_ produces a copolymer of 2HB and 3HB in *Escherichia coli*. Notably, the copolymer synthesized by PhaC_AR_ was found to be a block copolymer P(2HB-*b*-3HB)^19^.

Block copolymers are generally known to possess characteristic and useful properties, and used in the broad range of applications, such as elastomers^21^, drug delivery^22^ and lithography^23,24^. For this reason the biosynthesis of block PHAs has attracted great research interest^25^. A typical attempt to generate a block copolymer is to switch the monomer precursors in the medium during PHA production^26,27^. However, this strategy can lead to the generation of a polymer blend rather than a block copolymer because the extension of each polymer chain is presumably more rapid than the cultivation time^19,28^. Therefore, rapid and finely controlled feed switching is required^29^. Another obstacle toward block PHA biosynthesis is the difficulty in proving the block structure of the obtained polymer. Differences in the physical properties of the polymer, obtained using a switching strategy and the relevant polymer blend^30^, are not conclusive evidence of the block sequence. In contrast, we previously reported that PhaC_AR_ spontaneously synthesized P(2HB-*b*-3HB) from a mixture of 2HB and 3HB precursors in a medium without any external manipulation during cultivation. The block structure of P(2HB-*b*-3HB) was verified based on the microphase separation and solvent fractionation^19^. Thus, P(2HB-*b*-3HB) was the first structure-proven block PHA entirely composed of PHA monomer constituents, while PHAs connected with other types of polymers, such as polyethylene glycol^31,32^ and polyphosphate^33^, have been reported.

The aim of this study is to characterize the physical properties of P(2HB-*b*-3HB) and to compare them against a relevant random copolymer, P(2HB-*ran*-3HB). The P(2HB) homopolymer is a transparent and extensible material with an elongation at beak of 173%^34^. In contrast, P(3HB) is a stiff and brittle material due to its high crystallinity. Thus, the effect of combining P(2HB) (soft segment) and P(3HB) (hard segment) on the physical properties of the block polymers, particularly the soft segment-rich P(2HB-*b*-3HB), was of interest. As a result, it was demonstrated that P(2HB-*b*-3HB) can be processed into an elastomer-like material.

## Materials and methods

### Polymer production and analyses

*E. coli* JM109 chemical competent cells (Toyobo, Japan) were used for the transformation. The transformed cells harboring pBSP_Re_phaC_AR_pct^19^, which harbors the *phaC*_AR_ gene and the propionyl-CoA transferase gene from *Megasphaera elsdenii* under the control of the *R. eutropha phb* operon promoter, was grown on LB plate (tryptone 10 g/L, yeast extract 5 g/L, sodium chloride 10 g/L) containing 100 mg/L ampicillin and 15 g/L agar at 30 °C for 12 h. The seed culture was prepared using 1.5 mL LB medium containing 100 mg/L ampicillin at 34 °C for 12 h. The cells were used to inoculate the main culture of 100 mL LB medium containing 2% glucose, 100 mg/L ampicillin and different concentrations of sodium (*R,S*)-3HB and sodium (*R,S*)-2HB at 34 °C for 48 h, where the temperature was used to promote the production of the P(2HB) segment^35^. pBSP_Re_phaC1STQKpct^19^ was also used under the same culture conditions. The intracellular polymer in lyophilized cells was extracted with chloroform at 60 °C for 48 h. The extracted polymer was passed through a polytetrafluoroethylene membrane (pore size 0.2 μm) and precipitated by adding excess hexane. The precipitant was rinsed with methanol. The polymer content and monomer composition were determined using ^1^H nuclear magnetic resonance (NMR) spectroscopy. ^1^H NMR analysis of the extracted polymer in CDCl_3_ was performed as previously described in the literature^19^. The molecular weight of the polymer was determined by size exclusion chromatography using polystyrene standards for calibration, as described previously in the literature^19^.

### Preparation of the P(2HB-*co*-3HB) solvent-cast films

Solvent-cast films of P(2HB-*co*-3HB) were prepared as follows. Approximately 250 mg of purified polymer was dissolved in 7 mL of chloroform. The solution was placed in a glass Petri dish, which was covered with aluminum foil with ten holes (Φ =  ~ 1 mm), and placed on a horizontal table at room temperature for 3 d to allow the solvent to evaporate. After this time, the obtained circular film was further dried in vacuo for 24 h to remove any residual solvent. The films were stored at room temperature for at least two weeks and then subjected to testing.

### Thermal properties analysis

Differential scanning calorimetry (DSC) of the films was recorded using a DSC3 + STAR^e^ system (Mettler Toledo) under the following conditions. Approximately 3 mg of polymer sample was placed under an atmosphere of nitrogen gas. The temperature was calibrated using indium and zinc. Thermograms were recorded using the following two heating cycles. In the first heating cycle, the sample was heated from − 50 to 210 °C at a rate of 20 °C/min, held at 210 °C for 2 min, rapidly cooled to − 50 °C, and then held at this temperature for 5 min. In the second heating cycle, the sample was heated from − 50 to 210 °C at a rate of 20 °C/min and then held at 210 °C for 2 min. The melting temperature (*T*_m_) and the enthalpies of melting (Δ*H*_m_) were calculated from the thermogram of the first cycle. The glass transition (*T*_g_) and crystallization (*T*_c_) temperatures were calculated from the second heating cycle, because the crystals in the polymer melt completely during the first heating and *T*_g_ and *T*_c_ of amorphous phase are clearly observed in the second heating. The degree of crystallinity of P(3HB) phase was calculated considering the melting enthalpy of 100% crystallized P(3HB) of 146 J/g^36^. The degree of crystallization of P(2HB) phase was roughly estimated based on the melting enthalpy of P(2HB)^37^ because the melting enthalpy of 100% crystallized P(2HB) has not been determined.

The annealing conditions were explored using DSC. The samples were treated in a DSC machine using the heating cycle shown in Supplementary Fig. S1. Then, the thermal properties were determined as mentioned above.

### Wide angle X-ray diffraction analysis

Wide angle X-ray diffraction (WAXD) was performed on the films at the BL-6A of the Photon Factory (Tsukuba, Japan) using a synchrotron X-ray radiation (*λ* = 1.50 Å). The films were sandwiched between Kapton films and were subjected to the WAXD experiments at 25 °C. The X-ray diffraction data were collected for 60 s using a Pilatus 100 k detector, and the obtained 2D diffraction profiles were circularly averaged to yield the 1D profiles. The diffraction angle (2*θ*) was calibrated based on the diffractions from silver behenate.

### Mechanical properties of the films

The tensile strength, Young’s modulus, and elongation at break of the films were determined using a tensile testing machine (EZ-test, Shimadzu Co., Japan), operated at a tensile speed of 10 mm/min. The tests were conducted at 25 °C, unless otherwise specified. Samples were cut from the films using a dumbbell-shaped cutter SDMP-1000-D (Dumbbell Co., Ltd, Japan), with a gauge length and width of 12 and 2 ± 0.1 mm, respectively. P(92 mol% 2HB-*ran*-3HB) was tested with a gauge length of 8 mm to avoid the deformation of the gripping area of the film. The Young’s modulus was calculated from the stress–strain curve from 0.05 to 0.25%. The contraction behavior of the film was determined as follows. The dumbbell-shaped film was elongated 10 mm, then taken out of the tensile testing machine before it fractured. The gauge length of the film was measured after 24 h.

### Thermal processing of the films

The solvent-cast films were annealed using a heat press machine (H300-01, AS ONE, Japan), without applying any pressure, to accelerate the crystallization. The films were annealed at 70 °C for 24 h, and then rapidly chilled on ice, subsequently held at − 20 °C for 5 min in a freezer, and then stored at room temperature. Subsequently, the film was treated at 120 °C for 3 h, and then rapidly chilled on ice, subsequently held at − 20 °C for 5 min, and then stored at room temperature. In this step, only the P(2HB) phase was melted and subsequently became amorphous or very low crystallinity state, while the crystalline state of the P(3HB) phase was maintained. The obtained films are referred to as melt-quenched films.

## Results

### Synthesis of P(2HB-*b*-3HB) and P(2HB-*ran*-3HB)

P(2HB-*b*-3HB) and P(2HB-*ran*-3HB) were synthesized in recombinant *E. coli* JM109 expressing PhaC_AR_ and PhaC1_Ps_STQK, respectively (Table 1). The monomer sequences of these copolymers were confirmed using ^1^H NMR (Supplementary Fig. S2). The interpretation of the resonances is described in detail in the literature^19^. In brief, the resonance of methine proton of 2HB units in P(2HB-*b*-3HB) (5.1 ppm) was ascribed to 2HB-2HB*-2HB triad sequence, indicating the block sequence of the polymer (Supplementary Fig. S2). The monomer composition of P(2HB-*b*-3HB) was observed to change depending on the concentration of the monomer precursors added to the medium.

**Table 1:** Synthesis of P(2HB-*b*-3HB) and P(2HB-*ran*-3HB) in *E. coli.*

PHA synthase	Monomer precursor concentration (g/L)		CDW(g/L)	Polymer production (g/L)	Polymer content (wt%)	Monomer composition (mol%)	
	2HB-Na	3HB-Na				2HB	3HB
PhaC_AR_	5.0	2.5	2.28 ± 0.09	0.269 ± 0.020	11.8 ± 0.4	39.2	60.8
	7.5	2.5	1.97 ± 0.15	0.239 ± 0.019	12.1 ± 0.2	51.9	48.1
	10	1.0	2.10 ± 0.28	0.241 ± 0.041	11.5 ± 1.1	88.0	12.0
PhaC1_Ps_STQK	10	2.5	0.49 ± 0.06	0.069 ± 0.011	14.1 ± 1.4	92.0	8.0

Data are the mean ± standard deviation of three independent tests.

*CDW* cell dry weight.

### Phase separation in the P(2HB-*b*-3HB) solvent-cast film

Phase separation in the P(2HB-*b*-3HB) films, which enables the block copolymers to exhibit their characteristic physical properties, was investigated via analysis of their thermal properties (Table 2, Supplementary Figs. S3 and S4). The P(88 mol% 2HB-*b*-3HB) solvent-cast film exhibited two *T*_g_ shifts at 3.5 and 25.7 °C, indicating the phase separation of P(3HB) and P(2HB). However, the observed *T*_g_, which could be ascribed to the P(2HB) phase (25.7 °C), was lower than that of the P(2HB) homopolymer (30 °C)^34^, suggesting a partial mixing of the P(2HB) and P(3HB) phases. The melting peak at 155.5 °C could be ascribed to the P(3HB) phase. The melting temperature was lower than that of pure P(3HB), which could be due to the mixing of the phases and/or constrained crystal size. No clear melting peak was observed at around 100 °C, which is the melting temperature of P(2HB)^34^. Subtle fluctuations in the thermogram were observed in the temperature range of 80–90 °C (Supplementary Fig. S3). These results indicate that the P(2HB) phase was slightly and imperfectly crystalized during solvent casting.

**Table 2:** Thermal properties of P(2HB-*b*-3HB) and P(2HB-*ran*-3HB) films.

Sample	*T*_m_ (°C)	ΔH_m_ (J/g)	Crystallinity of P(3HB) phase (%)	*T*_g_ (°C)	*T*_c_ (°C)
Solvent-cast P(88 mol% 2HB-*b*-3HB)	155.5	8.3	47	3.5, 25.7	ND
Annealed P(88 mol% 2HB-*b*-3HB)	99.9, 156.3	9.1, 8.0	46	3.4, 26.4	ND
Annealed and melt-quenched P(88 mol% 2HB-*b*-3HB)	141.8	12.4	71	3.7, 26.2	ND
Solvent-cast P(46 mol% 2HB-*b*-3HB)	162.1	47.1	60	4.9, 19.3	89.3
Annealed P(46 mol% 2HB-*b*-3HB)	164.8	42.7	54	4.6, 17.0	94.3
Annealed and melt-quenched P(46 mol% 2HB-*b*-3HB)	163.0	52.1	66	4.9, 17.2	92.3
Solvent-cast P(92 mol% 2HB-*ran*-3HB)	ND	ND		23.4	ND

The annealed films were incubated at 70 °C for 24 h in a heat press machine. The melting temperature (*T*_m_) and enthalpy of fusion (ΔH_m_) were determined in the first heating scan and the glass transition (*T*_g_) and crystallization temperatures (*T*_c_) were determined in the second heating scan. The melt-quenched films were incubated at 120 °C for 3 h and − 20 °C for 5 min. ND = not detected.

P(2HB-*ran*-3HB) exhibited no melting peaks in the first and second heating scans, indicating that the random copolymer is almost amorphous. In addition, the copolymer exhibited a single *T*_g_, situated between those of P(2HB) and P(3HB). This result was consistent with the copolymer having a random structure.

### Annealing of P(2HB-*b*-3HB) to accelerate its crystallization

P(2HB-*b*-3HB) solvent-cast films were annealed to accelerate the crystallization of the P(2HB) phase, and consequently, to promote phase separation. The annealing conditions (70 °C for 24 h) were chosen based on the isothermal crystallization of P(88 mol% 2HB-*b*-3HB) (Supplementary Table S1). The annealed P(88 mol% 2HB-*b*-3HB) film exhibited a melting peak at 99.9 °C (Table 2), indicating that P(2HB) phase was partly crystallized. The degree of crystallization of P(2HB) phase was low based on the ΔH_m_ value at P(2HB) melting temperature (8.9 J/g) compared to the reported value of P(2HB) (67.5 J/g)^37^. The second melting peak of P(88 mol% 2HB-*b*-3HB) at 156.3 °C was ascribed to the crystallization of the P(3HB) phase. After the melt-quenching treatment of the P(2HB) phase, the P(2HB) melting peak disappeared and P(3HB) melting was observed at a slightly lower temperature of 141.8 °C, with a greater ΔH_m_ value of 12.4 J/g, indicating that the P(2HB) phase became amorphous, whereas the P(3HB) phase remained crystalline. The subtle fluctuations in the thermogram of the solvent-cast film observed in the temperature range of 80–90 °C were not detected in the melt-quenched film (Supplementary Fig. S4), indicating the increase in the amorphous P(2HB) phase. The crystallinity of the P(3HB) phase in P(88 mol% 2HB-*b*-3HB) increased from 47 to 71% by the thermal treatments (Table 2). The melting peak of the crystalline P(2HB) was not observed for P(46 mol% 2HB-*b*-3HB) after the annealing process (Table 2), presumably because the presence of the P(3HB) phase slows the crystallization of the P(2HB) phase.

### Crystal structure analysis by X-ray diffraction

**Figure 1:**
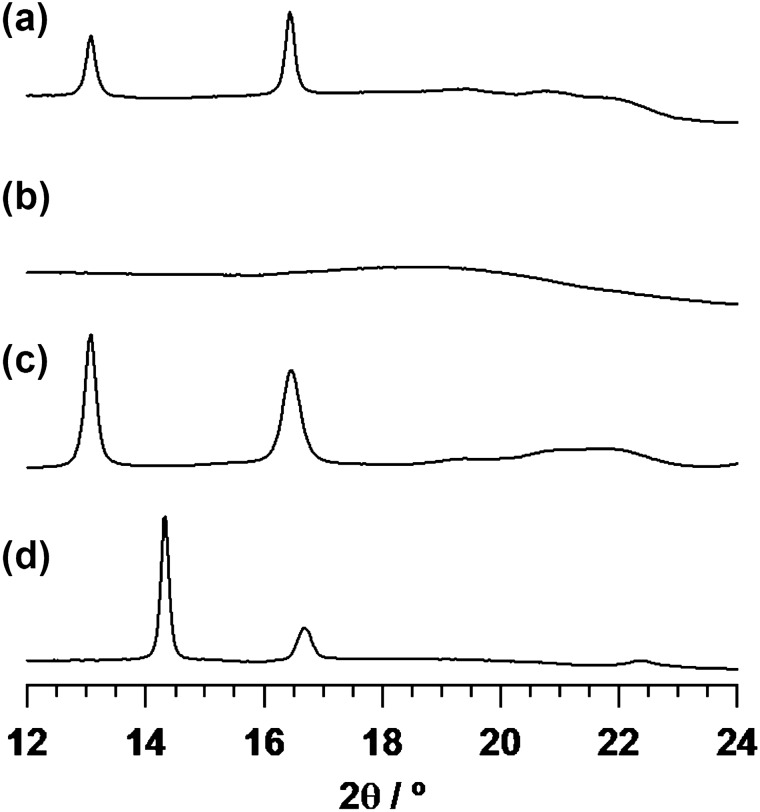
WAXD profiles of (**a**) P(88 mol% 2HB-*b*-3HB), (**b**) P(2HB-*ran*-3HB), (**c**) P(3HB), and (**d**) P(2HB) films. The diffraction peaks at 13.1 and 16.4° in (**a**) and (**c**) correspond to the orthorhombic P(3HB) crystal.

Wide-angle X-ray diffraction (WAXD) analysis was carried out to investigate the crystal structure of the melt-quenched P(88 mol% 2HB-*b*-3HB) film (Fig. 1). The WAXD profiles of the P(88 mol% 2HB-*b*-3HB) sample (Fig. 1a) and P(3HB) reference sample (Fig. 1c) showed clear diffraction peaks at 2*θ* (*λ* = 1.50 Å) of 13.1 and 16.4°, which correspond to the 020 and 110 reflections from the orthorhombic P(3HB) crystal^38^. Meanwhile, no peak corresponding to the P(2HB) crystal was observed in the P(88 mol% 2HB-*b*-3HB) film. As shown in Fig. 1d, the P(2HB) reference sample showed the diffraction peaks at 2*θ* of 14.3 and 16.7°. Although the exact crystal structure is not known, those diffractions are assignable to P(2HB) crystal according to previous report^39^. This confirmed that only the P(3HB) segment crystallized through the melt-quenching processing. There were no clear peaks in the WAXD profile of P(2HB-*ran*-3HB), indicating that the polymer was amorphous. These results were consistent with the thermal properties of the films (Table 2).

### Mechanical properties of the P(2HB-*b*-3HB) and P(2HB-*ran*-3HB) films

**Figure 2:**
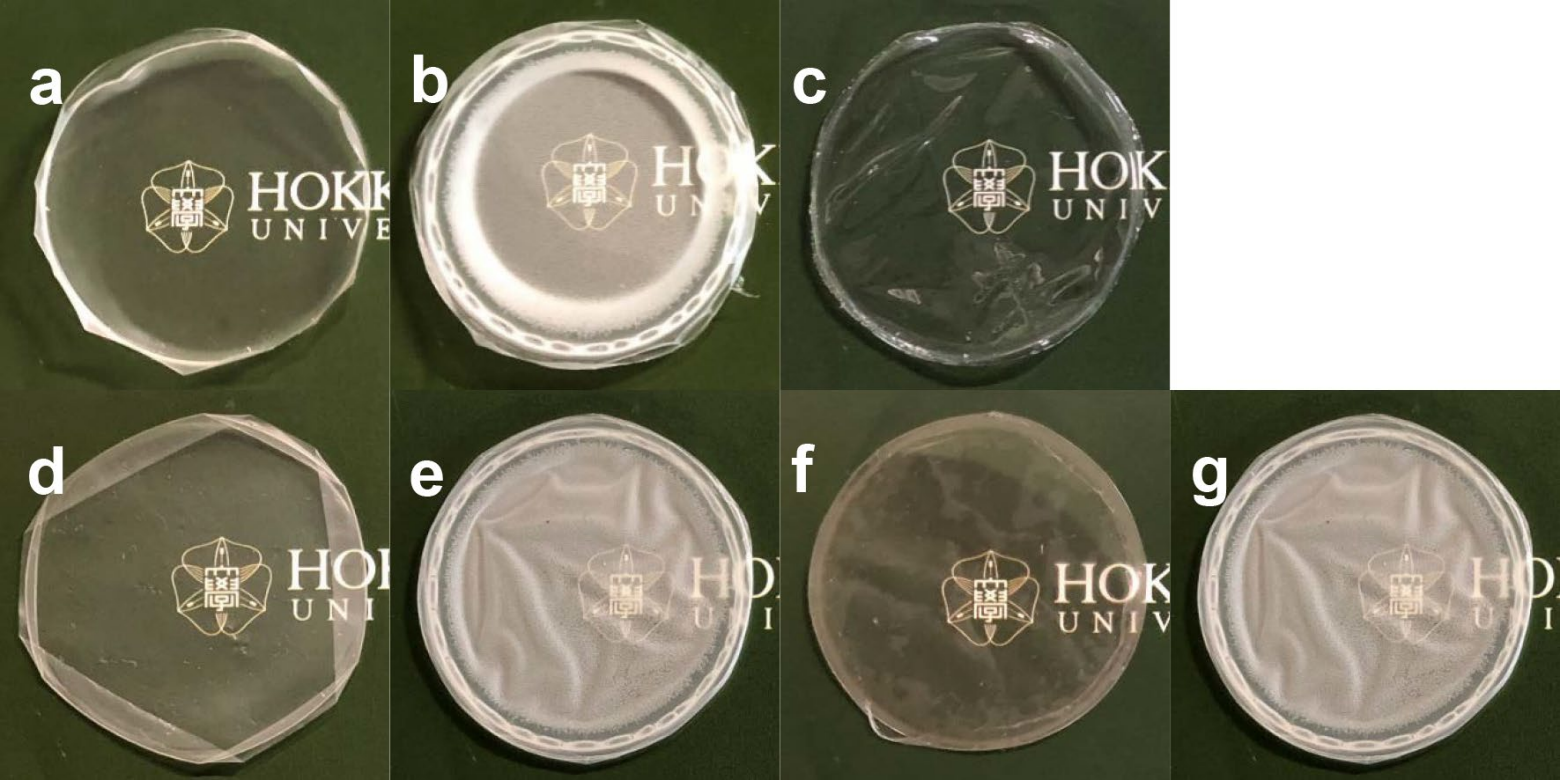
Films of the block and random copolymers of 2HB and 3HB. (**a**) solvent-cast film of P(88 mol% 2HB-*b*-3HB); (**b**) solvent-cast film of P(46 mol% 2HB-*b*-3HB); (**c**) solvent-cast film of P(92 mol% 2HB-*ran*-3HB); (**d**) film of P(88 mol% 2HB-*b*-3HB) after annealing at 70 °C for 24 h; (**e**) film of P(46 mol% 2HB-*b*-3HB) after annealing at 70 °C for 24 h; (**f**) annealed and melt-quenched film of P(88 mol% 2HB-*b*-3HB); (**g**) annealed and melt-quenched film of P(46 mol% 2HB-*b*-3HB). Diameter of the films is approximately 4.8 cm.

The appearances of the solvent-cast and thermally treated films are shown in Fig. 2. P(92 mol% 2HB-*ran*-3HB) was processed into a transparent and pliable film, as shown in Fig. 2c, whereas the P(88 mol% 2HB-*b*-3HB) and P(46 mol% 2HB-*b*-3HB) films were less pliable and transparent than the P(2HB-*ran*-3HB) film (Fig. 2a, b). The molecular weight of the polymers slightly decreased during the annealing process, probably due to the thermal decomposition. The molecular weight of P(92 mol% 2HB-*ran*-3HB) copolymer was lower than those of block copolymers (Table 3), because the random copolymer was synthesized by class II PhaC1_Ps_STQK. The molecular weight of PHAs synthesized by class II PHA synthase is known to be lower than that synthesized by class I enzymes^40–42^, but the mechanism is not understood at the molecular level.

**Table 3:** Mechanical properties and molecular weights of P(2HB-*ran*-3HB) and P(2HB-*b*-3HB).

Sample	Tensile strength	Young’s modulus	Yield point	Elongation at break	*M*_w_	*M*_w_/*M*_n_
	(MPa)	(MPa)	(MPa)	(%)	(× 10^5^)	
Solvent-cast P(88 mol% 2HB-*b*-3HB)	38.1 ± 2.8	690 ± 127	17.3 ± 2.1	266 ± 16	7.7	2.7
Annealed P(88 mol% 2HB-*b*-3HB)	31.4 ± 6.0	962 ± 82	28.0 ± 1.6	202 ± 77	5.7	2.2
Annealed and melt-quenched P(88 mol% 2HB-*b*-3HB)	31.3 ± 1.6	578 ± 101	16.8 ± 1.1	393 ± 39	5.8	2.0
Solvent-cast P(46 mol% 2HB-*b*-3HB)	30.8 ± 1.5	886 ± 198	ND	35 ± 13	6.0	2.1
Annealed P(46 mol% 2HB-*b*-3HB)	27.4 ± 1.7	916 ± 207	ND	21 ± 2	3.4	1.9
Annealed and melt-quenched P(46 mol% 2HB-*b*-3HB)	14.0 ± 0.5	457 ± 88	ND	44 ± 6	3.6	1.8
Solvent-cast P(92 mol% 2HB-*ran*-3HB)	6.7 ± 0.7	186 ± 46	ND	692 ± 17	0.70	1.8

*M*_w_ = weight-average molecular weight. *M*_n_ = number-average molecular weight.

**Figure 3:**
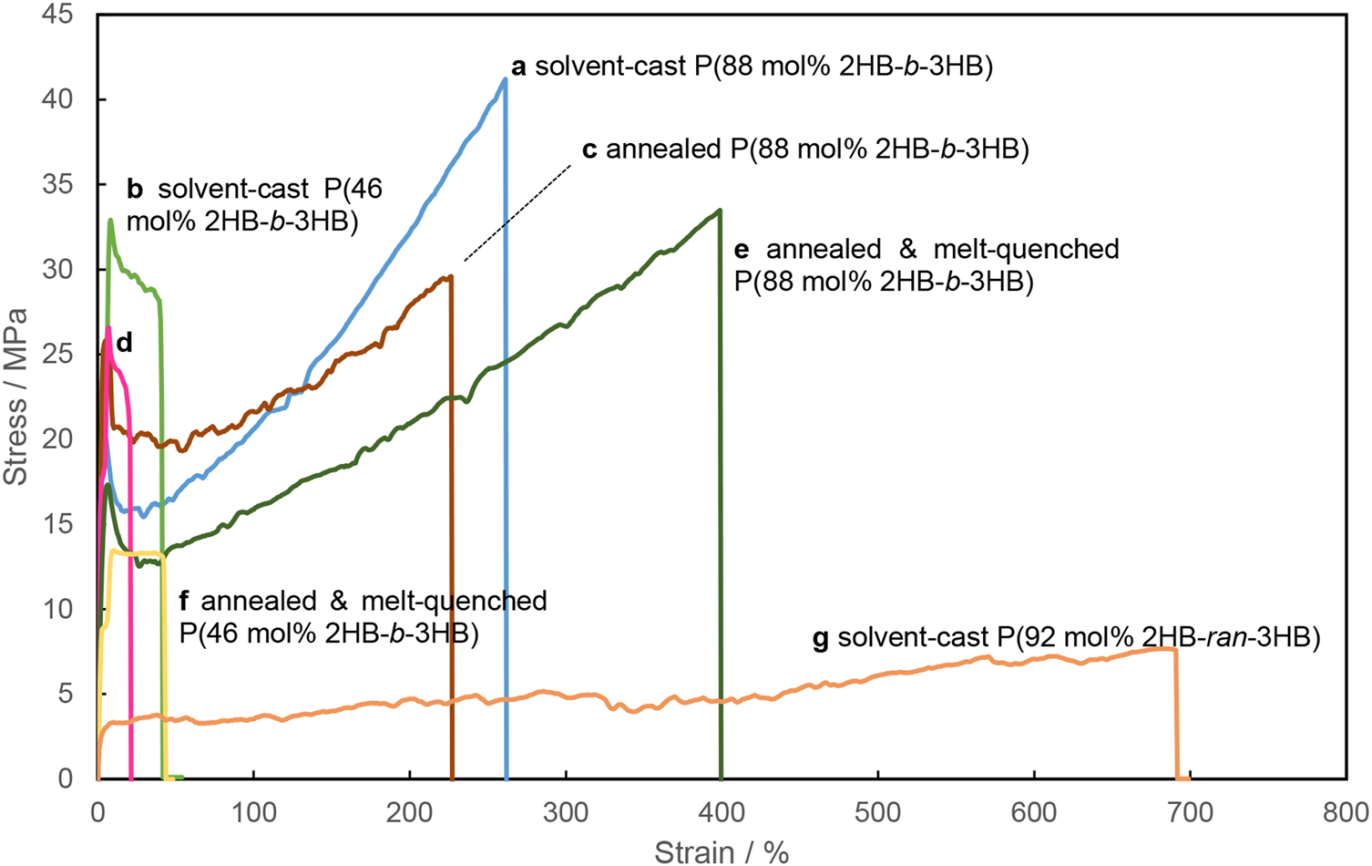
Stress–strain curves of the P(2HB-*b*-3HB) and P(2HB-*ran*-3HB) films. (**a)** solvent-cast P(88 mol% 2HB-*b*-3HB) (blue), (**b)** solvent-cast P(46 mol% 2HB-*b*-3HB) (light green), (**c)** annealed P(88 mol% 2HB-*b*-3HB) (brown), (**d)** annealed P(46 mol% 2HB-*b*-3HB) (pink), (**e**) P(88 mol% 2HB-*b*-3HB) annealed and melt-quenched (dark green), (**f**) P(46 mol% 2HB-*b*-3HB) annealed and melt-quenched (yellow), (**g**) solvent-cast P(92 mol% 2HB-*ran*-3HB) (orange). All tests were performed at 25 °C. The increasing stress during the period of elongation indicates the elastic properties of the films.

These films were subjected to uniaxial tensile testing, the results of which are shown in Fig. 3. P(92 mol% 2HB-*ran*-3HB) was stretched up to 692%, with only a small change observed in the stress (Fig. 3g). In contrast, solvent-cast P(88 mol% 2HB-*b*-3HB) exhibited a sharp increase in stress at the yield point, after which the stress increased during the period of elongation, as shown in Fig. 3a. The annealing of P(88 mol% 2HB-*b*-3HB) decreased its elongation to break and increased its Young’s modulus (Fig. 3c). The effect of annealing on the mechanical properties can be attributed to an increase in the crystallinity of P(2HB). In contrast, the melt-quenching of P(88 mol% 2HB-*b*-3HB) increased its elongation to break from 202 to 393% and decreased its Young’s modulus from 962 to 578 MPa (Fig. 3e, Table 3). These changes in the physical properties are presumably due to the decrease in the imperfect crystalline region of P(2HB) phase by the thermal treatments. P(46 mol% 2HB-*b*-3HB) exhibited hard and brittle properties, and it was observed that thermal treatment did not significantly change the mechanical properties of this film (Fig. 3b, d, and f). This result is presumably due to the higher fraction of the hard P(3HB) segment.

### Contraction behavior of P(2HB-*b*-3HB) film

A characteristic property of elastic materials is their contraction behavior after elongation. The melt-quenched P(85 mol% 2HB-*b*-3HB) film, which was prepared by the same conditions to P(88 mol% 2HB-*b*-3HB), was used for the contraction test. The original gauge length of the dumbbell-shaped film was 12 mm. The film was elongated 10 mm and taken out of the tensile testing machine before it fractured. The elongated film (22 mm in gauge length) exhibited no fast contraction behavior. However, the gauge length of the film was slowly reduced to 14 mm after 24 h incubation at 25 °C. This result indicates that P(2HB-*b*-3HB) exhibited a very slow contraction behavior. This phenomenon could be due to the *T*_g_ of the P(2HB) phase in P(2HB-*b*-3HB) (26.2 °C, Table 2), which is higher than the room temperature. This result indicates that P(2HB-*b*-3HB) has elastomer-like properties and that block PHAs have a potential to exhibit higher elasticity by proper molecular design of soft segments.

### Effect of temperature on the mechanical properties

**Figure 4:**
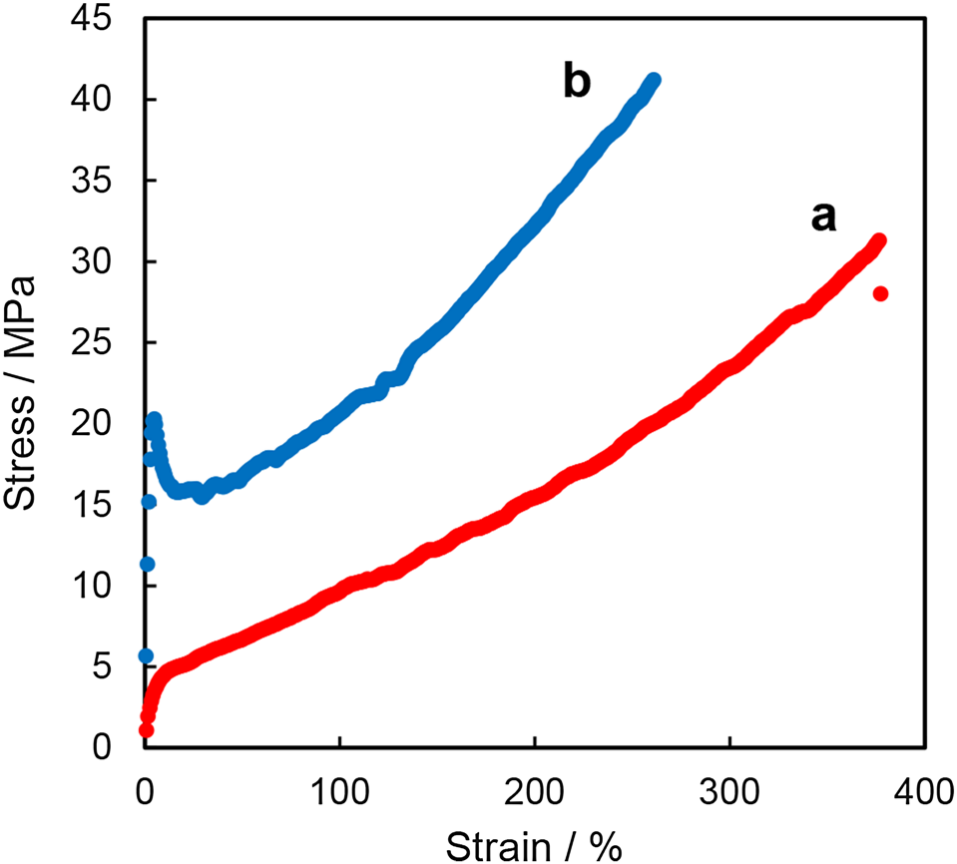
Stress–strain curves of the solvent-cast P(88 mol% 2HB-*b*-3HB) film above [32 °C (**a**) red] and/or below [25 °C (**b**) blue] its glass transition temperature. The yield point disappeared at 32 °C.

In general, the properties of polymers drastically change at the *T*_g_ because the amorphous phase becomes rubbery above this point. Therefore, the properties of P(2HB-*b*-3HB) should change at the *T*_g_ threshold if the amorphous P(2HB) phase contributes toward the stretching properties of the polymer. To examine this hypothesis, tensile tests were performed on P(88 mol% 2HB-*b*-3HB) below (25 °C) and above (32 °C) the *T*_g_ of P(2HB) (30 °C)^34^. As a result, no sharp increase in the stress at the yield point was observed at 32 °C, and the extension to break was considerably increased (375%) (Fig. 4). The results demonstrated that the elastic properties of the P(88 mol% 2HB-*b*-3HB) film can be attributed to rubbery amorphous P(2HB). In addition, the yield point observed at 25 °C was caused by the deformation of glassy amorphous P(2HB).

## Discussion

This study demonstrates that the monomer sequences of the PHA, whether random or block copolymers, have a critical effect on their physical properties. The stress–strain profiles of polymer films typically exhibit two characteristic curves; elastic deformation, in which the linear increase in stress is associated with the strain, and the material has not permanently deformed i.e. it has not been extended beyond the yield point, and plastic deformation, in which the strain increases without having any considerable effect on the stress and the material is no longer recoverable i.e. it has been extended beyond the yield point^43^. P(2HB-*ran*-3HB) showed a short region of elastic deformation in its initial stage of elongation. After the yield point was passed, plastic deformation of the film was observed until it fractured (Fig. 3g). The curve was similar to those of conventional PHA random copolymers that have pliable properties^44^. These properties of P(2HB-*ran*-3HB) can be due to its low crystallinity, as shown in DSC and WAXD analyses (Table 2 and Fig. 1).

In contrast, as shown in Fig. 3a, the P(88 mol% 2HB-*b*-3HB) film demonstrated a distinctly different stress–strain curve from that of the random copolymer (Fig. 3g), in that elastic deformation was observed until the film fractured, indicating an elastomer-like property. An increasing trend in the stress–strain curve has been reported for chemically crosslinked PHA^45^. Medium-chain-length (MCL) PHA with a terminal alkene in its side-chain, which was crosslinked via thiol-ene click chemistry, exhibited a stress–strain curve with an increasing trend, whereas the noncrosslinked control showed nearly constant stress during the period of elongation^45^. Based on the similarities in the stress–strain curves of the block *vs* random and crosslinked *vs* noncrosslinked polymers, it was presumed that P(2HB-*b*-3HB) has a noncovalent crosslinked structure. Noncovalent crosslinking points can be generated by the interactions between the polymer chains in the crystalline segment and/or glassy hard domains^46^. In P(88 mol% 2HB-*b*-3HB), the presence of P(3HB) crystals was demonstrated by DSC and WAXD analyses. The crystalline P(3HB) phase could serve as a noncovalent crosslinking point, contributing toward the elastic properties of the material. Polymer materials with a network of polymer chains connected via noncovalent linkages are referred to as supramolecular elastomers^46^. To the best of our knowledge, P(2HB-*b*-3HB) is the first elastomer-like PHA generated from a block sequence.

The distinctive properties of block copolymers can be attributed to the phase separation that occurs between each segment, meaning that the immiscibility of the segments is essential for the molecular design of block copolymers. This is particularly important for the molecular design of PHA block copolymers because some PHAs are miscible each other. As an example, P(3HB) and poly(3-hydroxyvalerate) [P(3HV)] were used in the first attempt to synthesize a block PHA. The polymer product containing P(3HB) and P(3HV) fractions had no distinct *T*_g_s, indicating the miscibility of these PHAs^26^. In contrast, the P(2HB-*b*-3HB) described in the present study possessed two *T*_g_s, which indicates the immiscibility of the P(2HB) and P(3HB) phases in the polymer. Therefore, the use of a P(2HB) segment possesses dual benefit: the generation of a homo-block sequence, and immiscibility with P(3HB). The next important step is the structural analysis of the phase separation in P(2HB-*b*-3HB). A sea-island structure in 3HB-rich P(2HB-*b*-3HB) was previously observed by atomic force microscopy^19^. However, the observation of phase separation in the 2HB-rich P(2HB-*b*-3HB) via atomic force microscopy is currently unsuccessful, partly due to the low crystallinity. Further studies are needed to clarify the structure of the phase separation in 2HB-rich block copolymers.

The thermal processing of P(2HB-*b*-3HB) was found to be effective in increasing the elongation to break of the polymer. The annealing process was intended to promote the phase separation of the P(2HB) and P(3HB) phases. In fact, the *T*_g_ ascribed to the P(2HB) phase was slightly increased upon annealing (Table 2). The decrease in the elongation at break of the annealed film was in good agreement with the increase in the crystallinity of the film. In the subsequent melt-quenching process, a treatment temperature of 120 °C was selected as this was in between the melting temperatures of P(2HB) (100 °C) and P(3HB) (~ 170 °C). As expected, only the P(2HB) crystal phase was converted into an amorphous phase during this process. The melt-quenched film exhibited a greater elongation to break. These results indicate the importance of thermal processing to enhance the elastic properties of polymers.

In conclusion, P(88 mol% 2HB-*b*-3HB) was found to be an elastomer-like material on the ground of its elastic deformation during elongation and slow contraction behavior after elongation. In contrast, P(92 mol% 2HB-*ran*-3HB) was stretchable but not elastic, similar to conventional PHA random copolymers. The crystalline P(3HB) phase in P(2HB-*b*-3HB) plays an important role in the elasticity of the polymer. The low elasticity of P(2HB-*b*-3HB) could be due to the high *T*_g_ of the soft P(2HB) segment. However, the findings in this study demonstrate the potential of block PHAs as elastic materials, whose elasticity can be improved by the molecular design of soft segments. Biodegradability of P(2HB) and relevant copolymers is currently under investigation. The cytotoxicity and biocompatibility assessment of these polymers also remains unachieved.

## Data availability

All data generated or analyzed during this study are included in this published article (and its Supplementary Information file).

## Supplementary Information

Supplementary Information.
