# Supplementary material for: Artificial polyhydroxyalkanoate poly[2-hydroxybutyrate-block-3-hydroxybutyrate] elastomer-like material
Source: Sci Rep. 2021 Nov 17;11:22446. doi: 10.1038/s41598-021-01828-9 (PMC8599675; doi:10.1038/s41598-021-01828-9)
Supplement: Supplementary file 1 — Supplementary Information. [file 41598_2021_1828_MOESM1_ESM.pdf]

Supplementary information

for

Artificial polyhydroxyalkanoate poly[2-hydroxybutyrate-block-3-hydroxybutyrate] elastomer-like material

Yuki Kageyama, Hiroya Tomita, Takuya Isono, Toshifumi Satoh and Ken’ichiro Matsumoto*

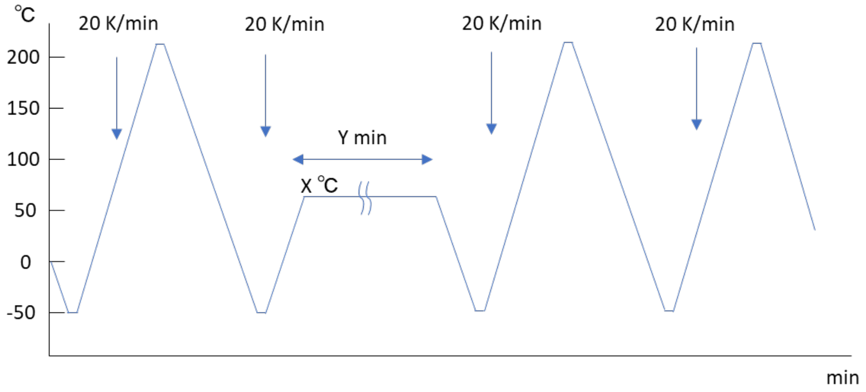

Figure S1. Heating and cooling cycles for annealing of the films, where X = 50 or 70 and Y = 720.

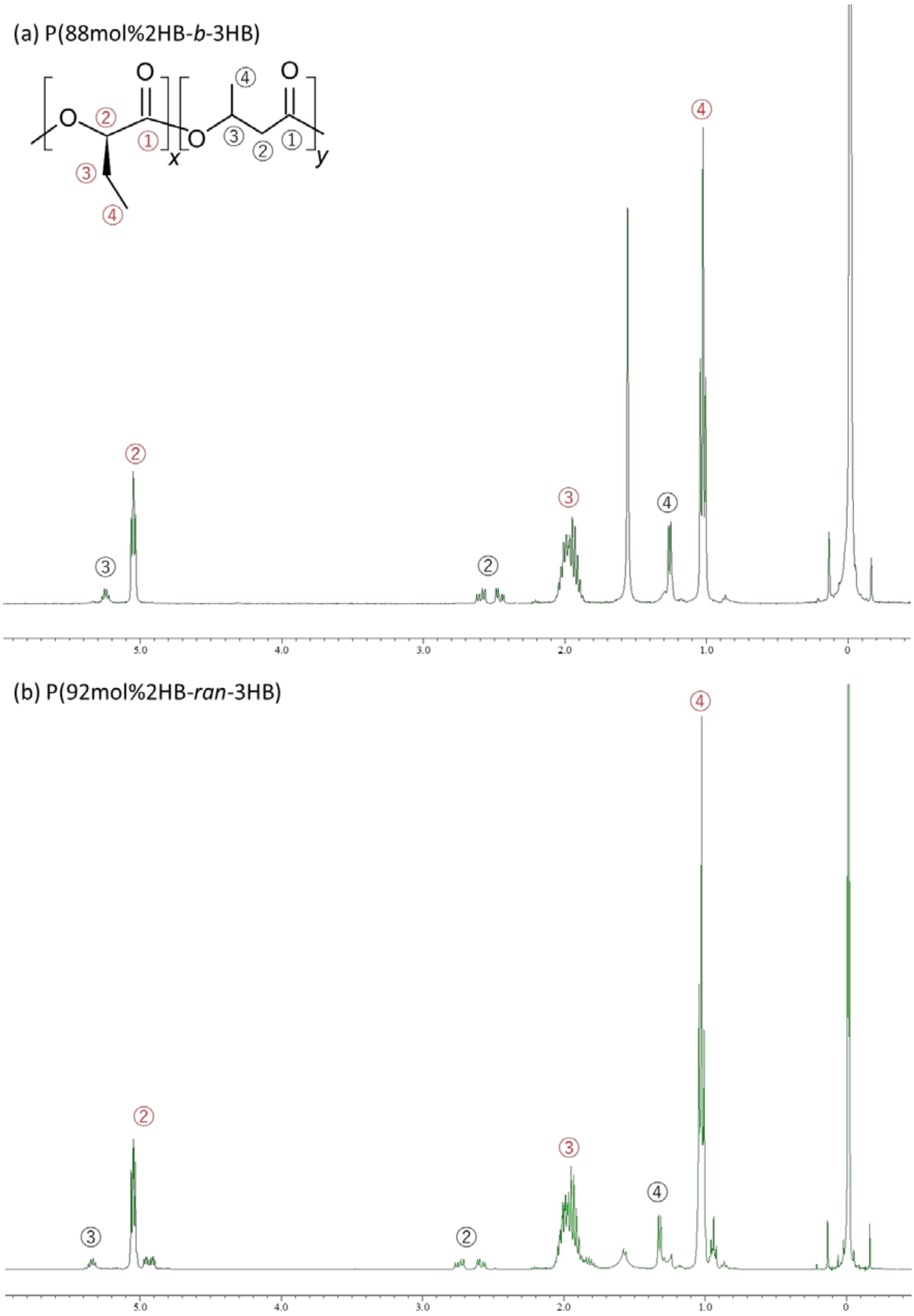

Figure S2. ^{1}H NMR spectra of the P(2HB-*co*-3HB) polymers synthesized in *E. coli*. (a) P(88 mol% 2HB-*b*-3HB) synthesized using PhaC$_{AR}$ and (b) P(92 mol% 2HB-*ran*-3HB) synthesized using PhaC1$_{Ps}$STQK.

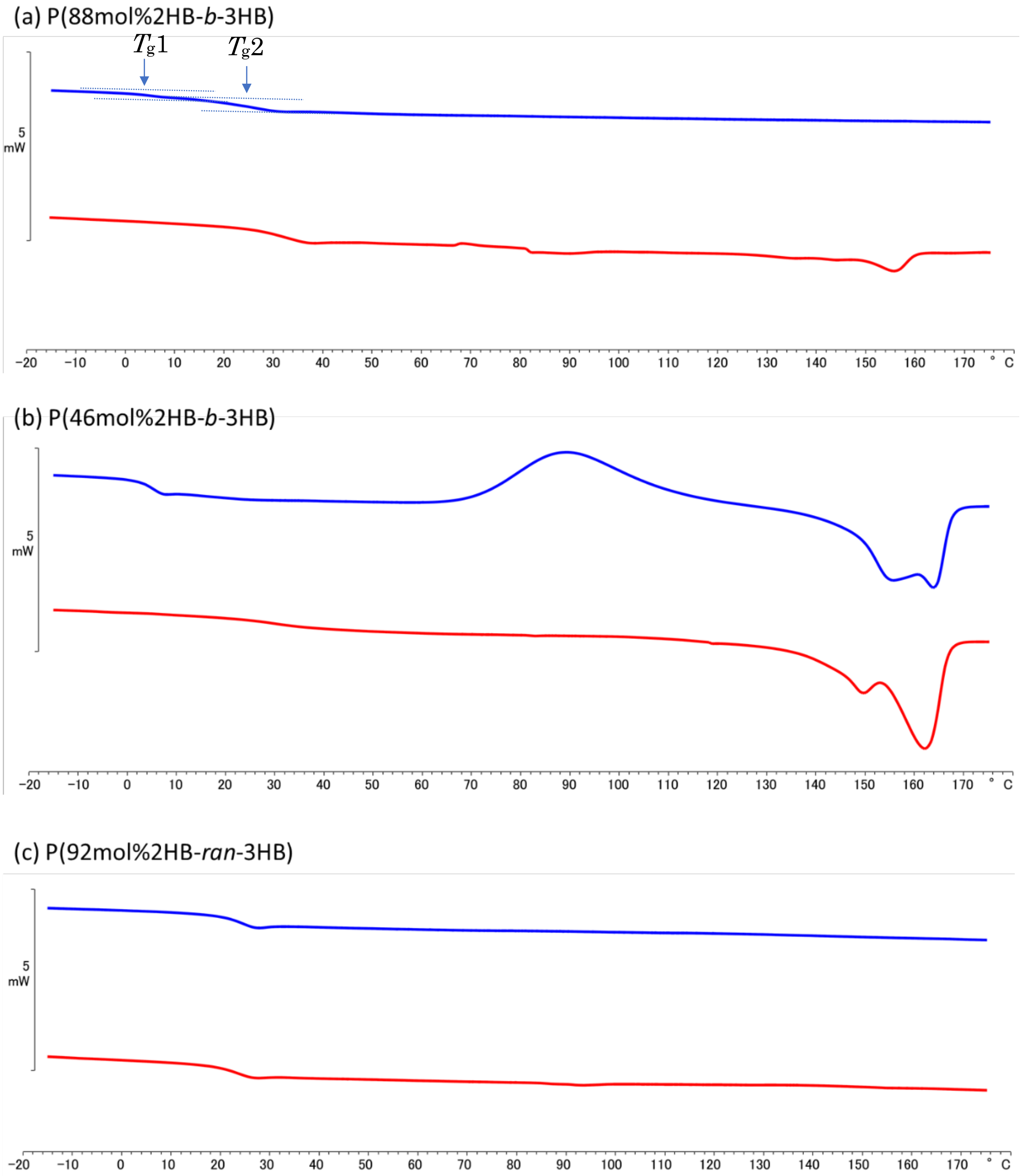

Figure S3. DSC thermograms of solvent-cast films of the P(2HB-*co*-3HB) polymers. First (red) and second (blue) heating scans were recorded for (a) P(88 mol% 2HB-*b*-3HB), (b) P(46 mol% 2HB-*b*-3HB), and (c) P(92 mol% 2HB-*ran*-3HB).

Table S1. Exploration of the annealing conditions of the P(88 mol% 2HB-*b*-3HB) film for promoting crystallization

<table>
<tr><th rowspan="2">Annealing temperature (°C)</th><th rowspan="2">Annealing time (h)</th><th colspan="2">T_m(°C)</th><th colspan="2">ΔH_m(J/g)</th></tr>
<tr><th>P(2HB)</th><th>P(3HB)</th><th>P(2HB)</th><th>P(3HB)</th></tr>
<tr><td>-</td><td>-</td><td>ND</td><td>155.5</td><td>ND</td><td>8.25</td></tr>
<tr><td>50</td><td>12</td><td>91.9</td><td>159.16</td><td>4.75</td><td>1.02</td></tr>
<tr><td>70</td><td>12</td><td>100.2</td><td>159.16</td><td>2.7</td><td>11.2</td></tr>
<tr><td>50</td><td>6</td><td rowspan="2">95.6</td><td rowspan="2">148.9</td><td rowspan="2">13.8</td><td rowspan="2">10.7</td></tr>
<tr><td>70</td><td>6</td></tr>
<tr><td>70</td><td>6</td><td>100.2</td><td>149.8</td><td>2.58</td><td>11.7</td></tr>
<tr><td>70</td><td>24</td><td>100.5</td><td>148.8</td><td>3.04</td><td>11.6</td></tr>
</table>

Annealing was performed in a DSC machine.

(a) P(88 mol% 2HB-*b*-3HB)

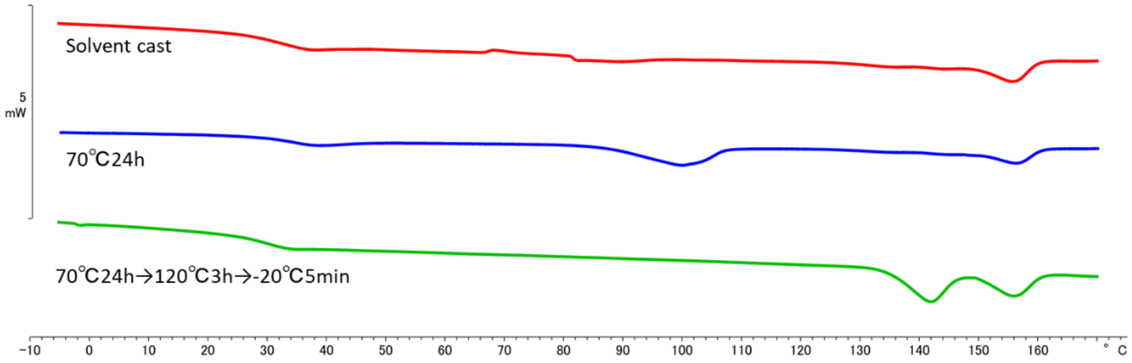

(b) P(46 mol% 2HB-*b*-3HB)

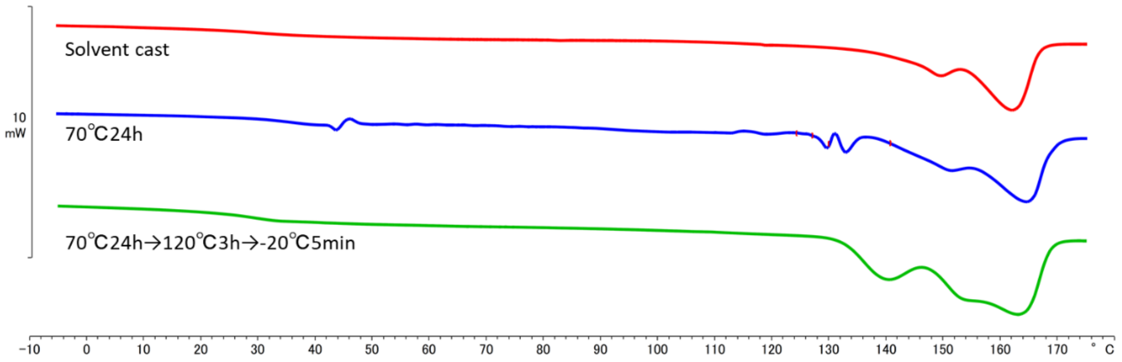

Figure S4. DSC thermograms of annealed P(88 mol% 2HB-*b*-3HB) (a) and P(46 mol% 2HB-*b*-3HB) (b) in a heat press machine.
